# Supplementary material for: Structural snapshots of V/A-ATPase reveal the rotary catalytic mechanism of rotary ATPases
Source: Nat Commun. 2022 Mar 8;13:1213. doi: 10.1038/s41467-022-28832-5 (PMC8904598; doi:10.1038/s41467-022-28832-5)
Supplement: Supplementary file 1 — Supplementary Information [file 41467_2022_28832_MOESM1_ESM.pdf]

Supplementary Information

Structural snapshots of V/A-ATPase reveal the rotary catalytic mechanism of rotary ATPases.

Authors: J. Kishikawa[1,2]†, A. Nakanishi[1,3]†, A. Nakano[1]†, S. Saeki[1], A. Furuta[1], T. Kato[2], K. Mistuoka[3], and K. Yokoyama[1]*.

Affiliations: [1]Department of Molecular Biosciences, Kyoto Sangyo University, Kamigamo-Motoyama, Kita-ku, Kyoto 603-8555, Japan;

[2]Institute for Protein Research, Osaka University, 3-2 Yamadaoka, Suita, Osaka 565-0871 Japan;

[3]Research Center for Ultra-High Voltage Electron Microscopy, Osaka University, 7-1, Mihogaoka, Ibaraki, Osaka, 567-0047 Japan.

†J.K, A. Nakanishi., and A. Nakano contributed equally to this work

*Corresponding author. Email: yokoken@cc.kyoto-su.ac.jp

Supplementary note

Catalytic state of nucleotide bound to AB_{closed}

In the V_{3nuc} structure, the EM density of the γ-phosphate of ATP in AB_{closed} is clearer than that in the V_{prehyd} structure (Figs 3 and Supplementary Figure 7). This difference is due to the length of the dwell time of the nucleotide in the catalytic site in the two different structures. The weak density of the γ-phosphate in the AB_{closed} of V_{prehyd} is likely due to the release of the cleaved phosphate during the slower hydrolysis reaction that occurs with ATPγS as a substrate. Since the ATP-binding dwell time is negligible under ATP-saturated conditions, V_{3nuc} structures likely reflect the structures during and/or just after ATP hydrolysis in AB_{closed}. In fact, γ-phosphate of ATP bound to the AB_{closed} of state1-2 of V_{3nuc} appears to be separated from β-phosphate (Supplementary Figure 10). In addition, the density of the γ-phosphate of ATP bound to AB_{closed} in V_{2nuc}, which has a longer dwell time for ATP, is also weak (Figs. 3a and Supplementary Figure 7a). Taken together, we conclude that the ATP-like density at the catalytic site of AB_{closed} of V_{3nuc} is ADP and phosphate following hydrolysis of ATP.

Supplementary Figures

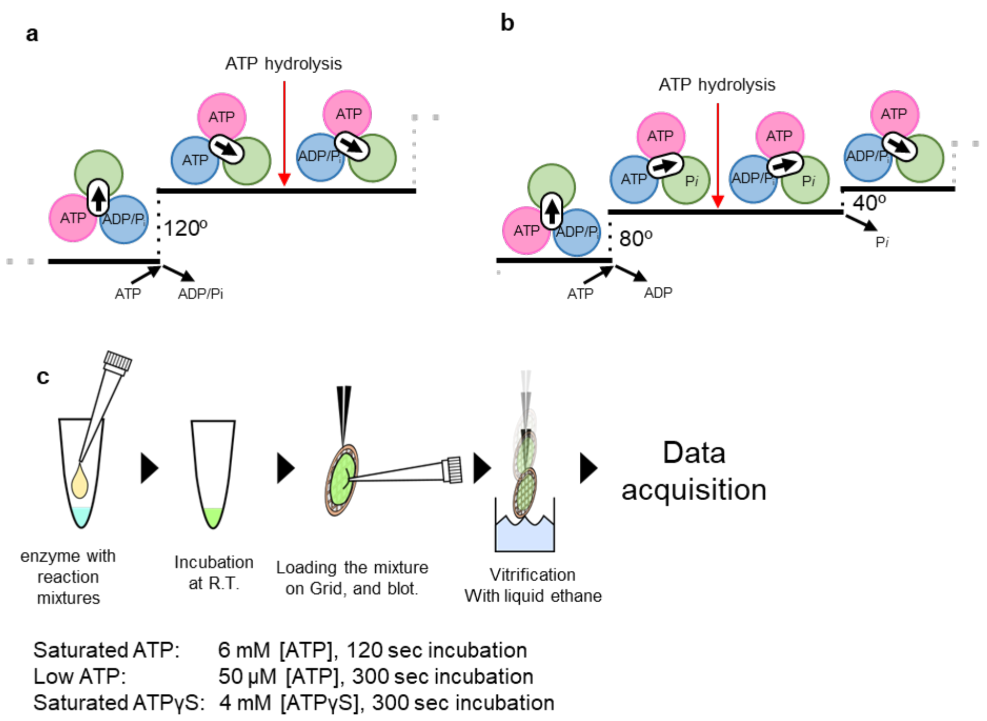

Supplementary Figure 1. Chemo-mechanical cycle of rotary ATPases. (**a, b**) The proposed chemo-mechanical cycle of V_1-ATPase[3] and F_1-ATPase[27]. **(c)** Experimental set up for grid preparation under different reaction conditions.

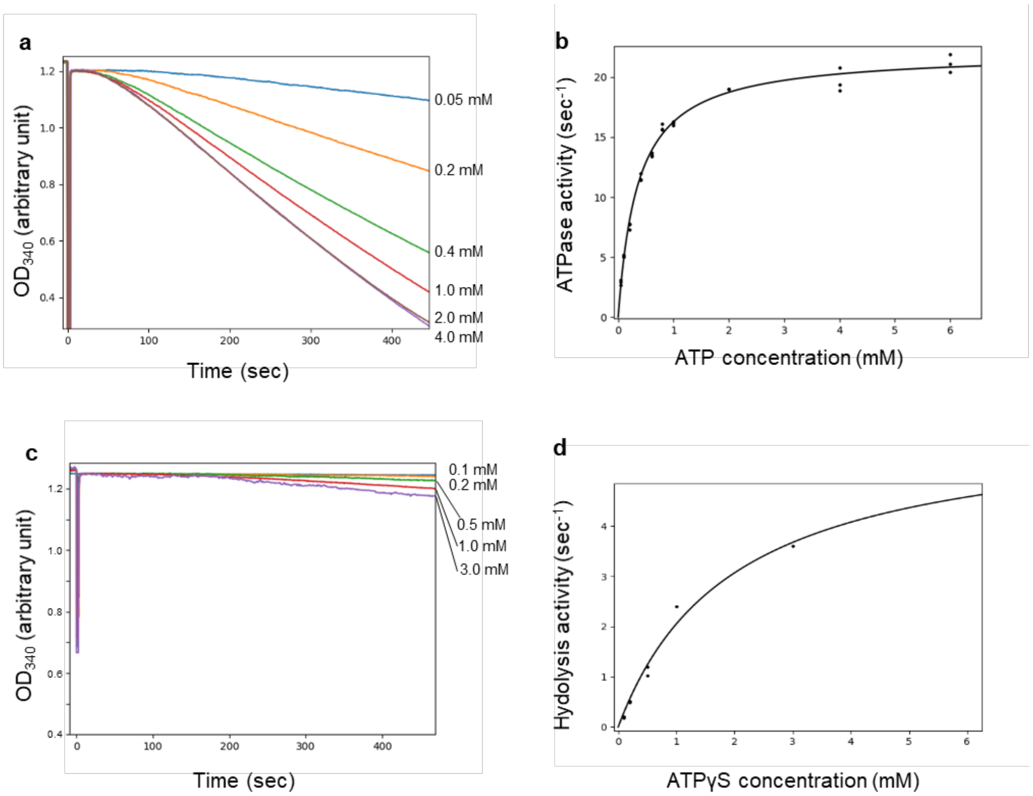

Supplementary Figure 2. Enzymatic properties of V/A-ATPase for cryo-gird preparation. **a**, ATP hydrolysis of nucleotide-free V/A-ATPase incorporated into nanodisc (*nd*-V/A-ATPase) at various ATP concentrations. **b**, *S vs V* plot for ATP hydrolysis by the nucleotide-free *nd*-V/A-ATPase. **c**, ATPγS hydrolysis of nucleotide-free *nd*-V/A-ATPase at various ATPγS concentrations. **d**, *S vs V* plot for ATPγS hydrolysis by the nucleotide-free *nd*-V/A-ATPase. The activities of the *nd*-V/A-ATPase were measured by the enzyme coupling assay described in the Method Details.

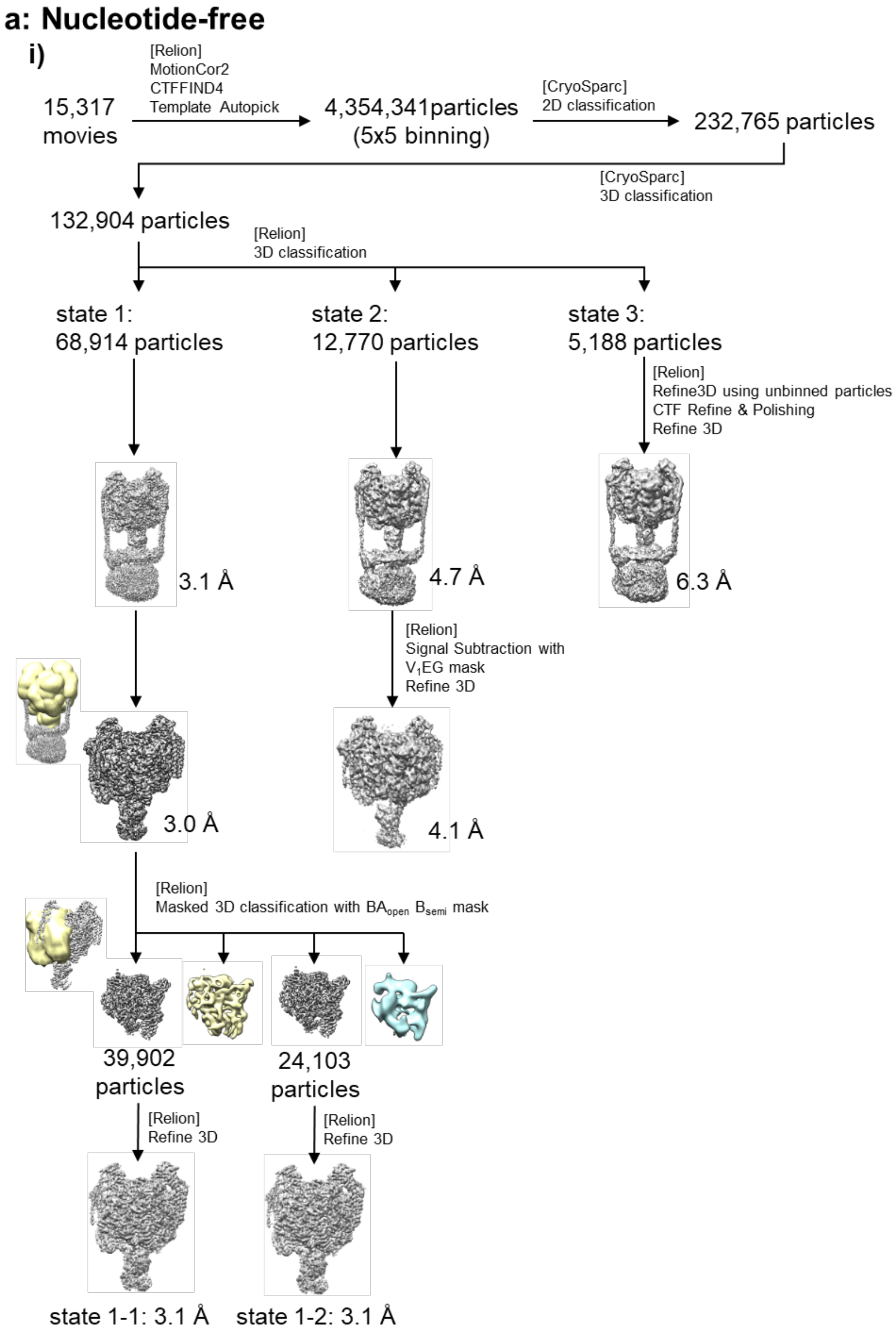
a: Nucleotide-free
i)
[Relion]
MotionCor2
CTFFIND4
Template Autopick
15,317 movies
4,354,341particles (5x5 binning)
[CryoSparc]
2D classification
232,765 particles
[CryoSparc]
3D classification
132,904 particles
[Relion]
3D classification
state 1:
68,914 particles
state 2:
12,770 particles
state 3:
5,188 particles
[Relion]
Refine3D using unbinned particles
CTF Refine & Polishing
Refine 3D
3.1 Å
4.7 Å
6.3 Å
[Relion]
Signal Subtraction with
V_1EG mask
Refine 3D
3.0 Å
4.1 Å
[Relion]
Masked 3D classification with BA_{open} B_{semi} mask
39,902 particles
24,103 particles
[Relion]
Refine 3D
[Relion]
Refine 3D
state 1-1: 3.1 Å
state 1-2: 3.1 Å

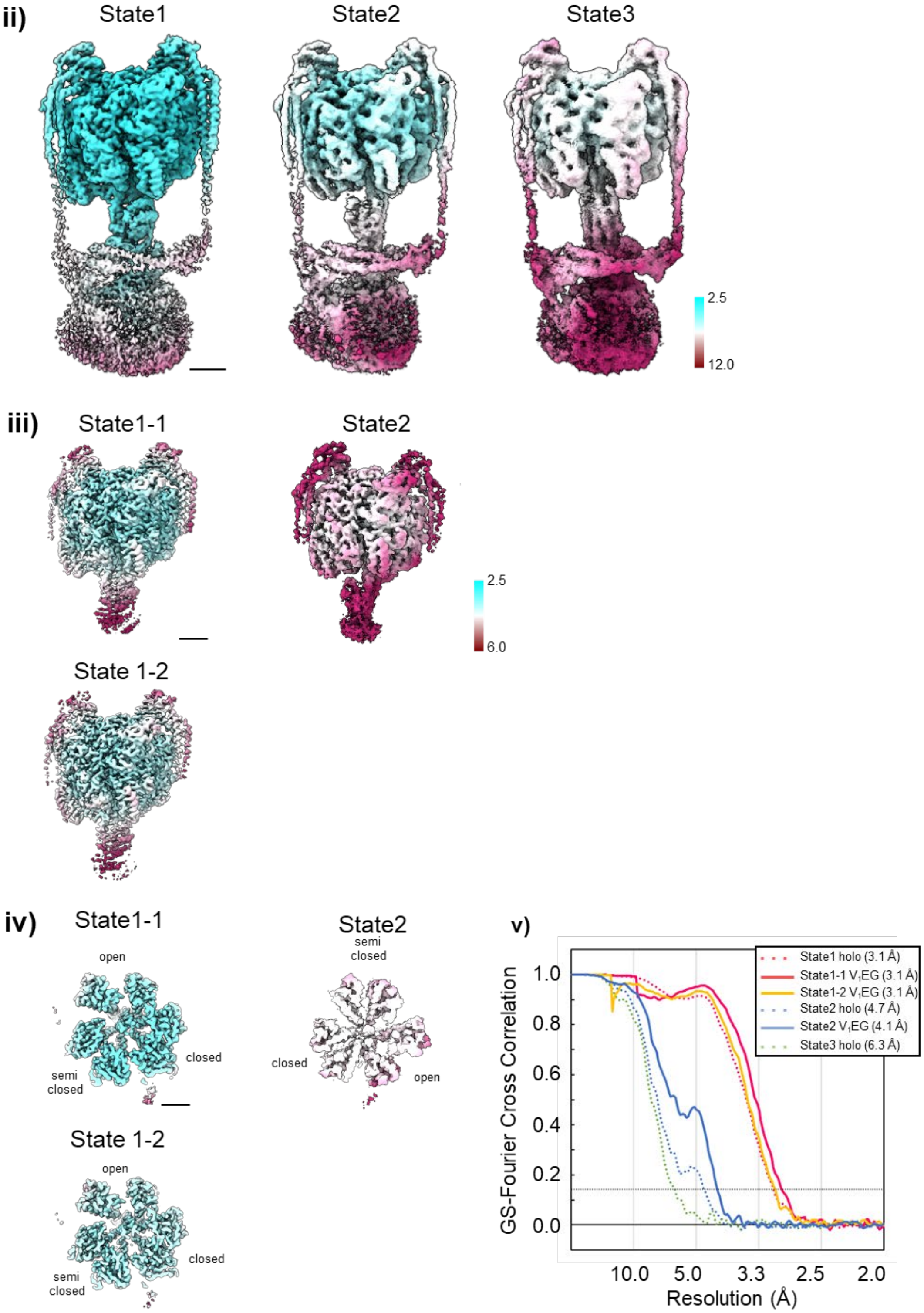

ii)
State1
State2
State3
2.5
12.0
iii)
State1-1
State2
2.5
6.0
State 1-2
iv)
State1-1
open
closed
semi
closed
State 1-2
open
closed
semi
closed
State2
semi
closed
closed
open
v)
State1 holo (3.1 Å)
State1-1 V1EG (3.1 Å)
State1-2 V1EG (3.1 Å)
State2 holo (4.7 Å)
State2 V1EG (4.1 Å)
State3 holo (6.3 Å)
GS-Fourier Cross Correlation
1.0
0.8
0.6
0.4
0.2
0.0
10.0
5.0
3.3
2.5
2.0
Resolution (Å)

b: Saturated ATP

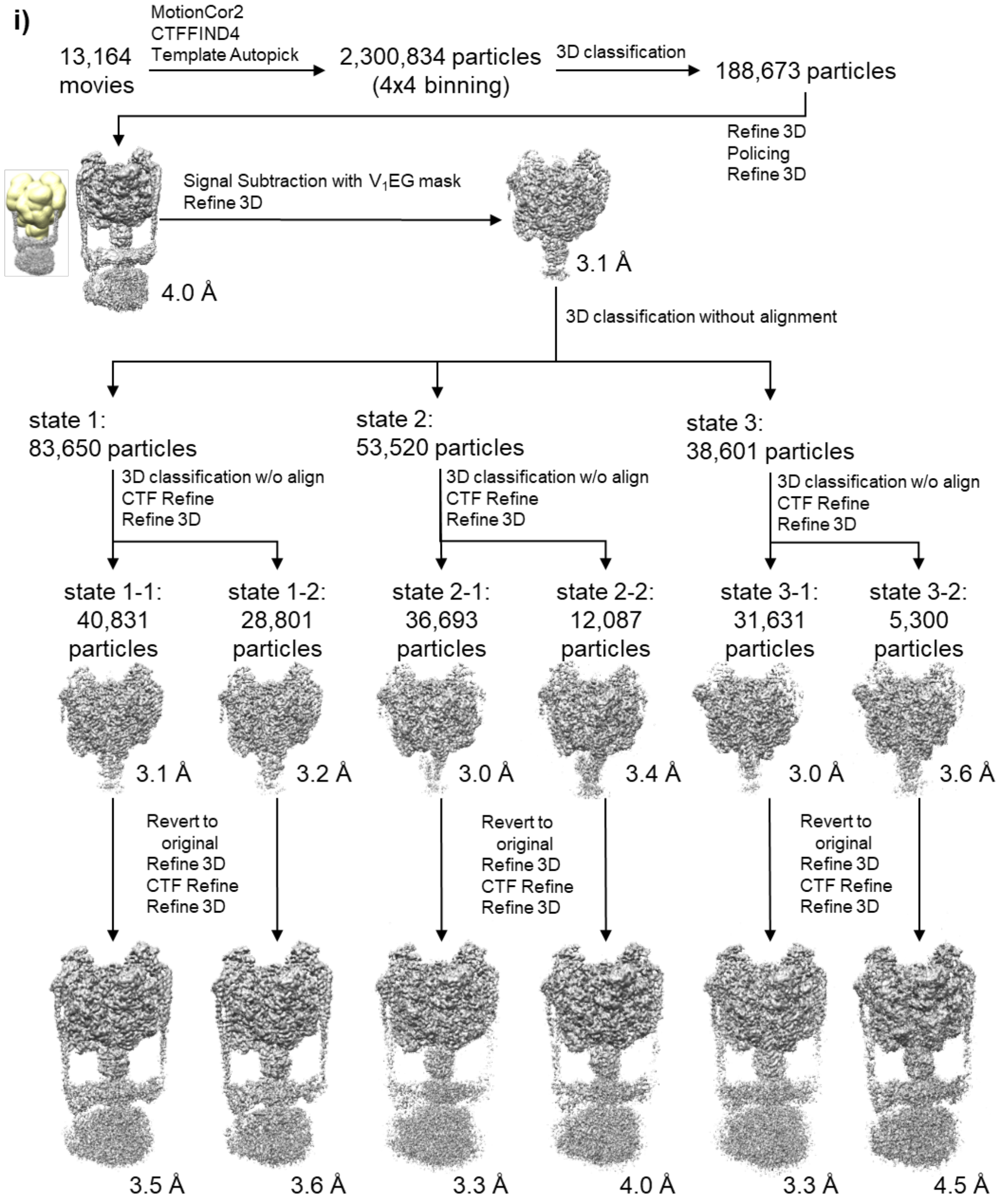

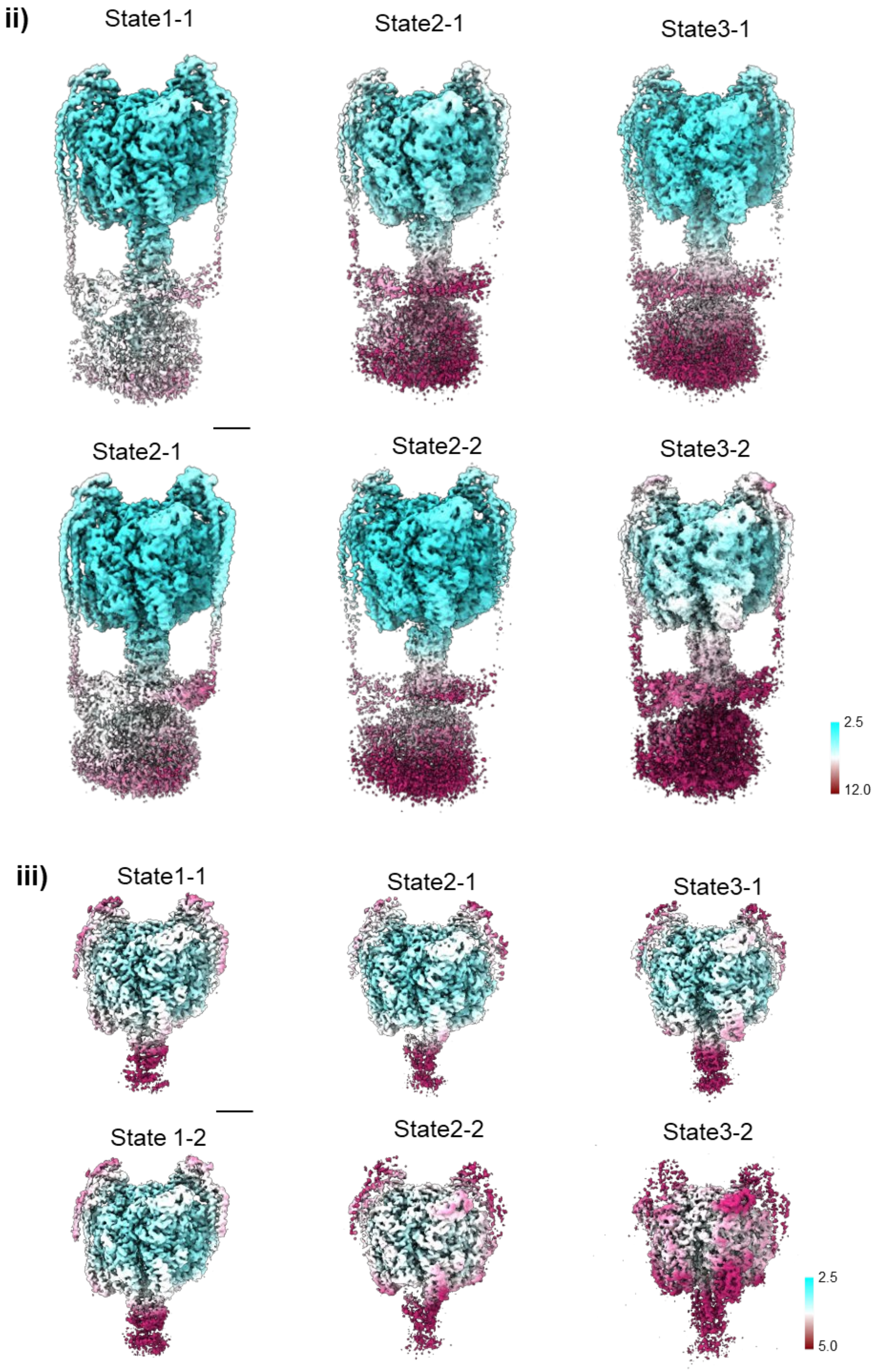

ii)
State1-1
State2-1
State3-1
State2-1
State2-2
State3-2
2.5
12.0
iii)
State1-1
State2-1
State3-1
State 1-2
State2-2
State3-2
2.5
5.0

iv)

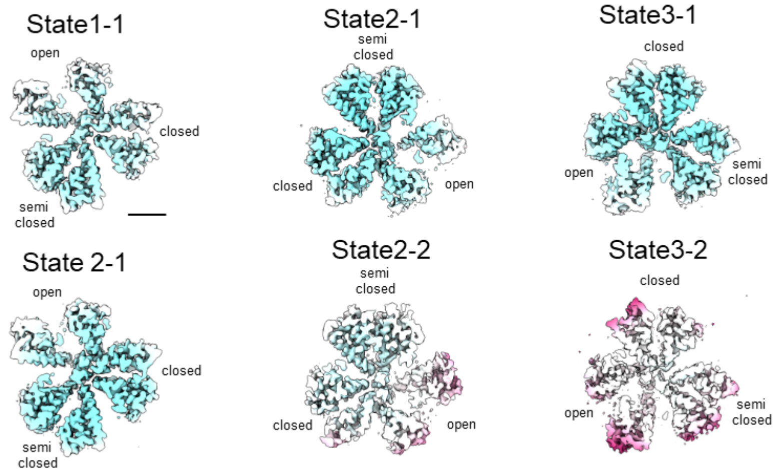
State1-1
open
closed
semi
closed
State2-1
semi
closed
closed
open
State3-1
closed
open
semi
closed
State 2-1
open
closed
semi
closed
State2-2
semi
closed
closed
open
State3-2
closed
open
semi
closed

v)

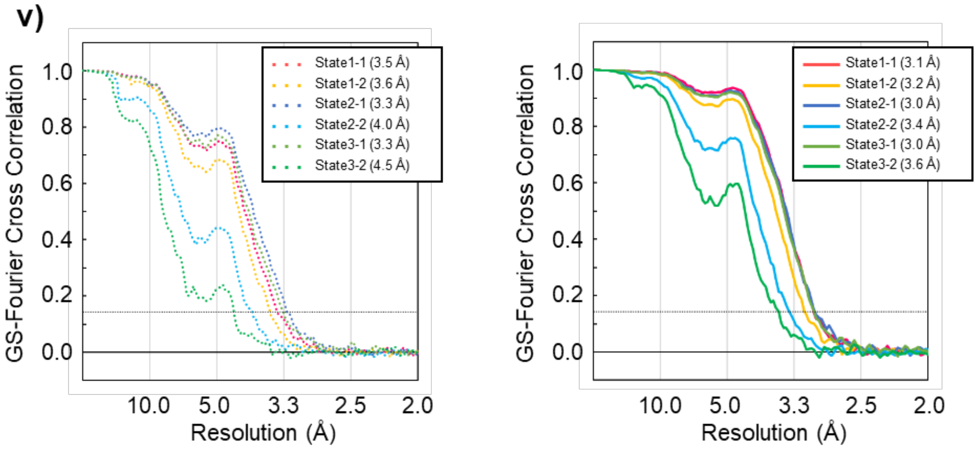
GS-Fourier Cross Correlation
1.0
0.8
0.6
0.4
0.2
0.0
10.0
5.0
3.3
2.5
2.0
Resolution (Å)
State1-1 (3.5 Å)
State1-2 (3.6 Å)
State2-1 (3.3 Å)
State2-2 (4.0 Å)
State3-1 (3.3 Å)
State3-2 (4.5 Å)
State1-1 (3.1 Å)
State1-2 (3.2 Å)
State2-1 (3.0 Å)
State2-2 (3.4 Å)
State3-1 (3.0 Å)
State3-2 (3.6 Å)

2

c: Awaiting ATP binding

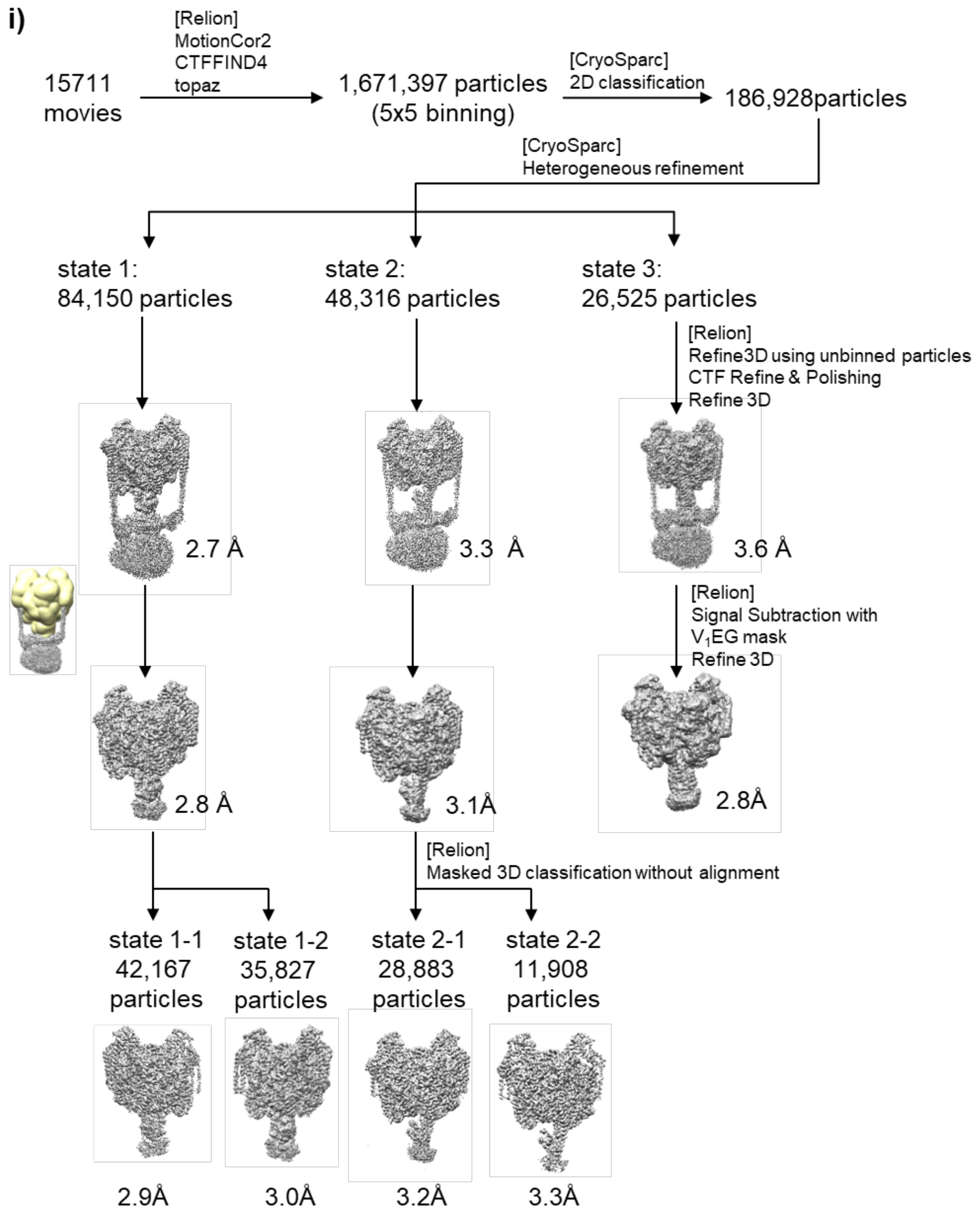

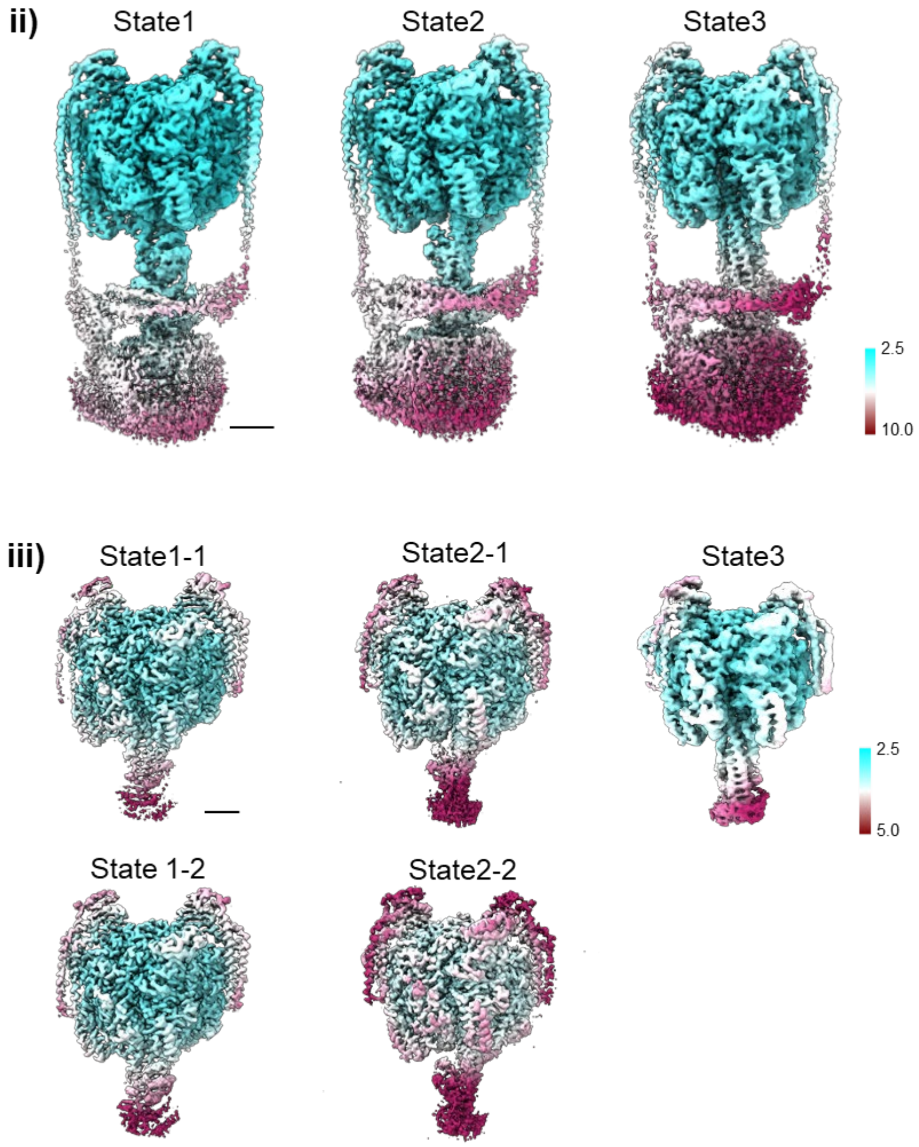
ii)
State1
State2
State3
2.5
10.0
iii)
State1-1
State2-1
State3
2.5
5.0
State 1-2
State2-2

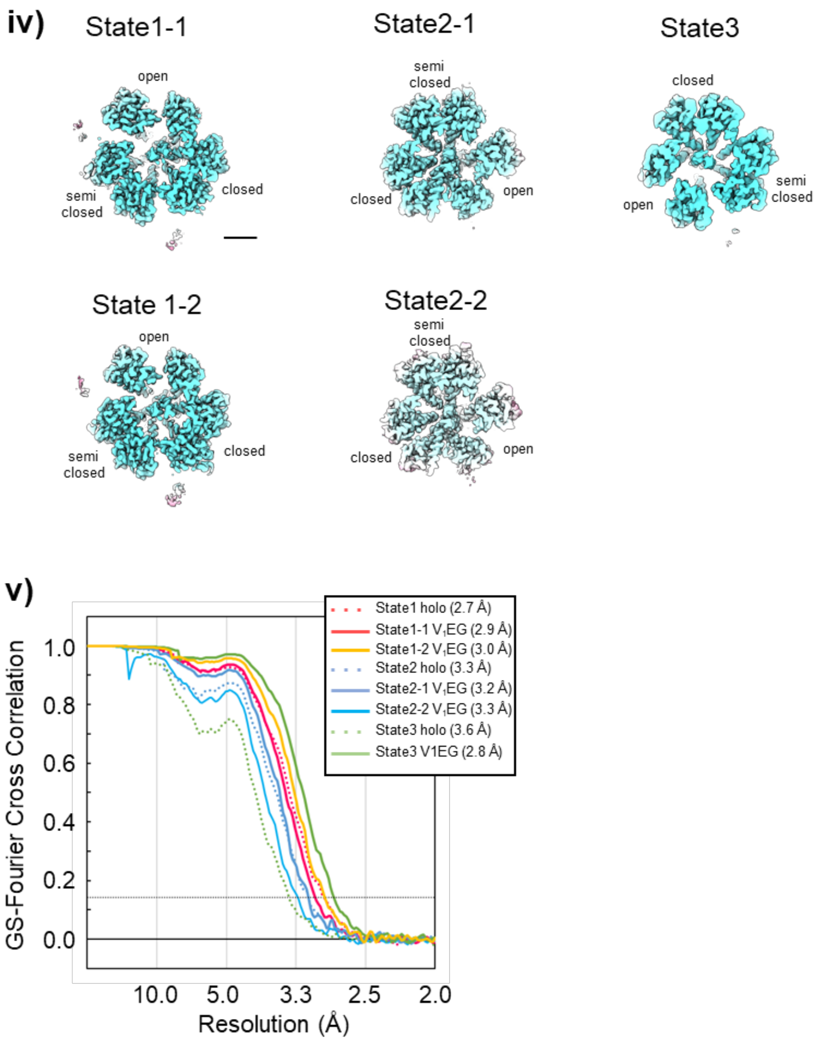

iv)
State1-1
open
semi closed
closed
State2-1
semi closed
closed
open
State3
closed
open
semi closed
State 1-2
open
semi closed
closed
State2-2
semi closed
closed
open
v)
State1 holo (2.7 Å)
State1-1 V1EG (2.9 Å)
State1-2 V1EG (3.0 Å)
State2 holo (3.3 Å)
State2-1 V1EG (3.2 Å)
State2-2 V1EG (3.3 Å)
State3 holo (3.6 Å)
State3 V1EG (2.8 Å)
GS-Fourier Cross Correlation
1.0
0.8
0.6
0.4
0.2
0.0
10.0
5.0
3.3
2.5
2.0
Resolution (Å)

2

d: saturated ATPγS

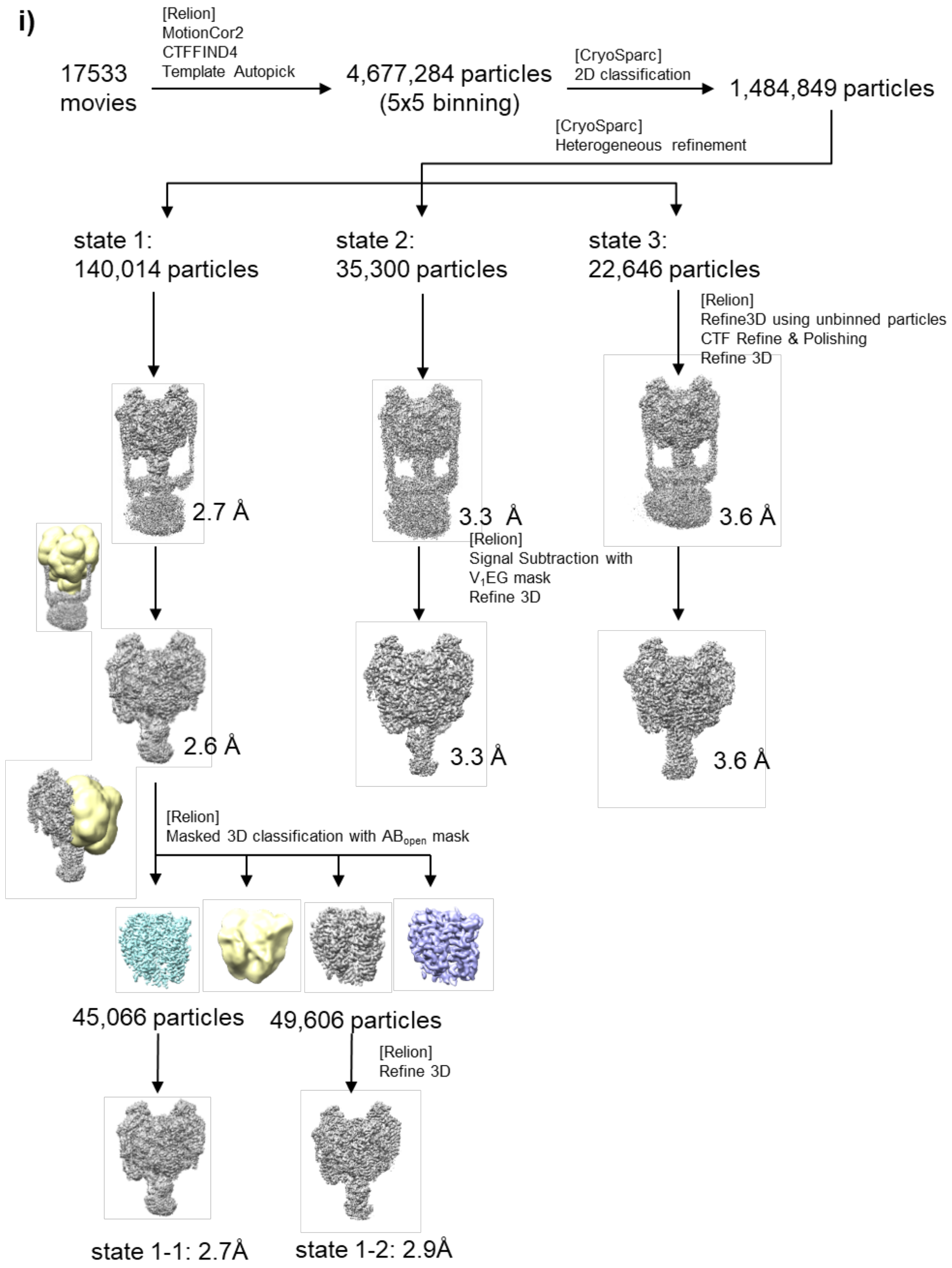

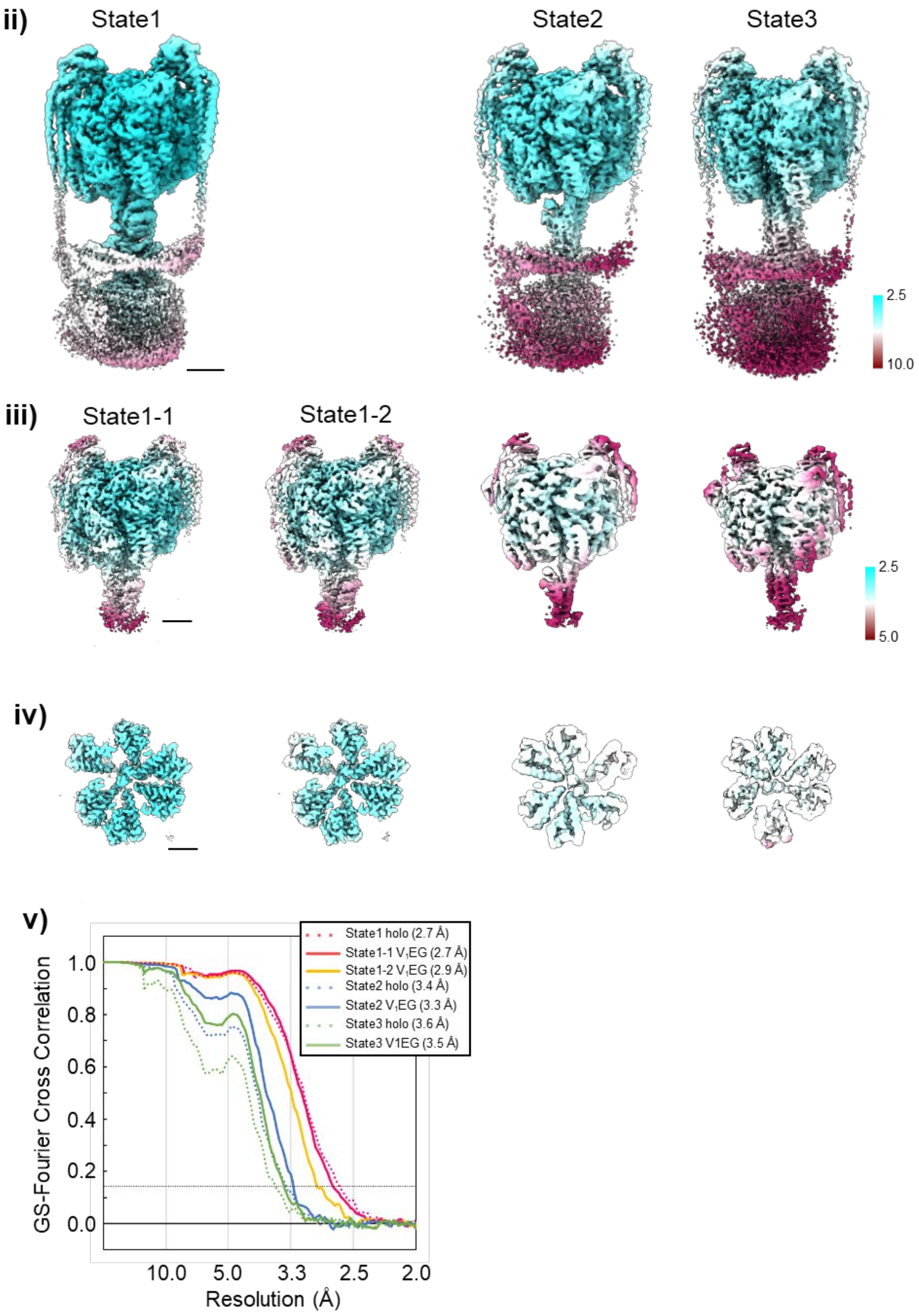

ii)
State1
State2
State3
2.5
10.0
iii)
State1-1
State1-2
2.5
5.0
iv)
v)
State1 holo (2.7 Å)
State1-1 V1EG (2.7 Å)
State1-2 V1EG (2.9 Å)
State2 holo (3.4 Å)
State2 V1EG (3.3 Å)
State3 holo (3.6 Å)
State3 V1EG (3.5 Å)
GS-Fourier Cross Correlation
1.0
0.8
0.6
0.4
0.2
0.0
10.0
5.0
3.3
2.5
2.0
Resolution (Å)

Supplementary Figure 3. Flow charts describing image acquisition and structural analysis allowing reconstitution of the 3D structures of Nucleotide-free V/A-ATPase (a), at a saturating ATP concentration (b), at ATP waiting condition (c), and at a saturated ATPγS concentration (d). i) Flow charts describing image acquisition and structural analysis allowing reconstitution of the 3D structures of V/A-ATPase under each condition. ii) Cryo-EM density maps of *holo* V/A-ATPase. iii) Side views of the cryo-EM density maps of V_1EG. iv) Cross sections of the V_1EG maps viewed from the cytosolic side. The maps are colored according to local resolution as indicated in the key. v) Gold standard Fourier shell correlation (FSC) curves for *holo* V/A-ATPase (dotted lines) and V_1EG (solid lines), using FSC = 0.143 for resolution criterion. Scale bar is 30 Å.

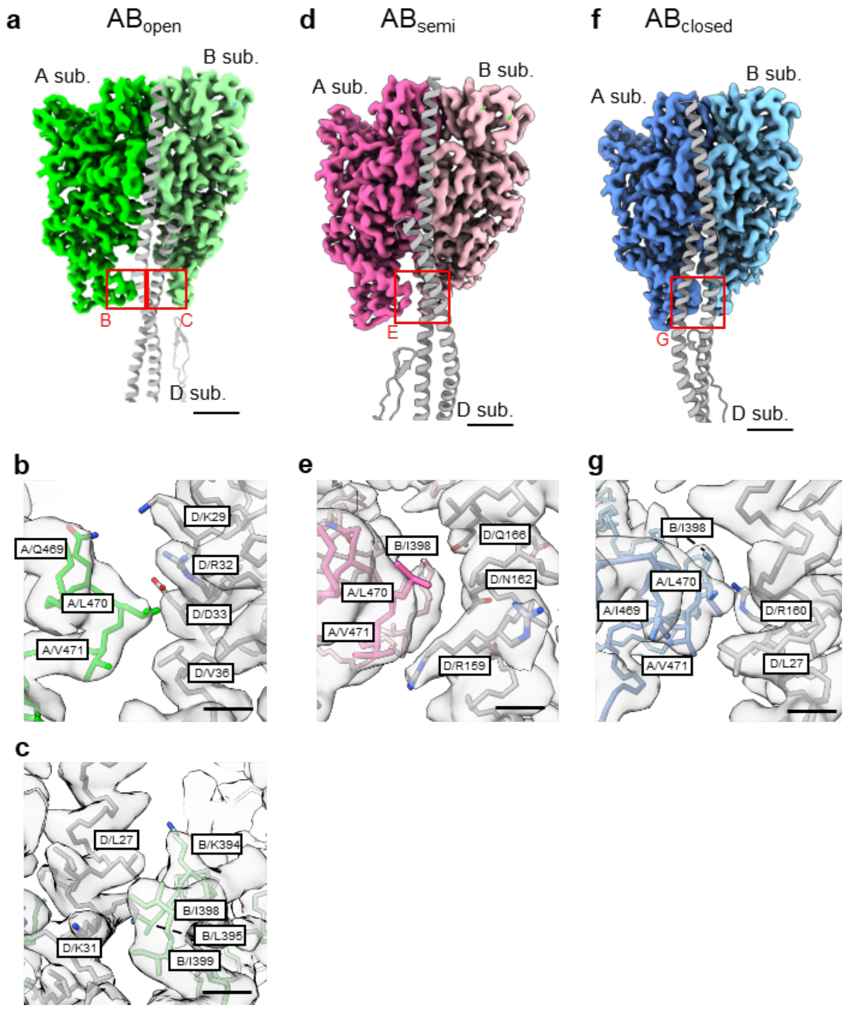

Suuplementary Figure 4. Interactions of DF with each AB dimer in $V_{nucfree}$. (a, d, f) The positioning of DF subunits relative to each AB dimer. AB dimers and DF subunits are shown in surface and ribbon representation, respectively. The scale bar is 15 Å. Red squares indicate the interacting interfaces of the CHB and D subunit and are shown enlarged in the lower panels **(b, c, e, g)**. The scale bar is 4 Å.

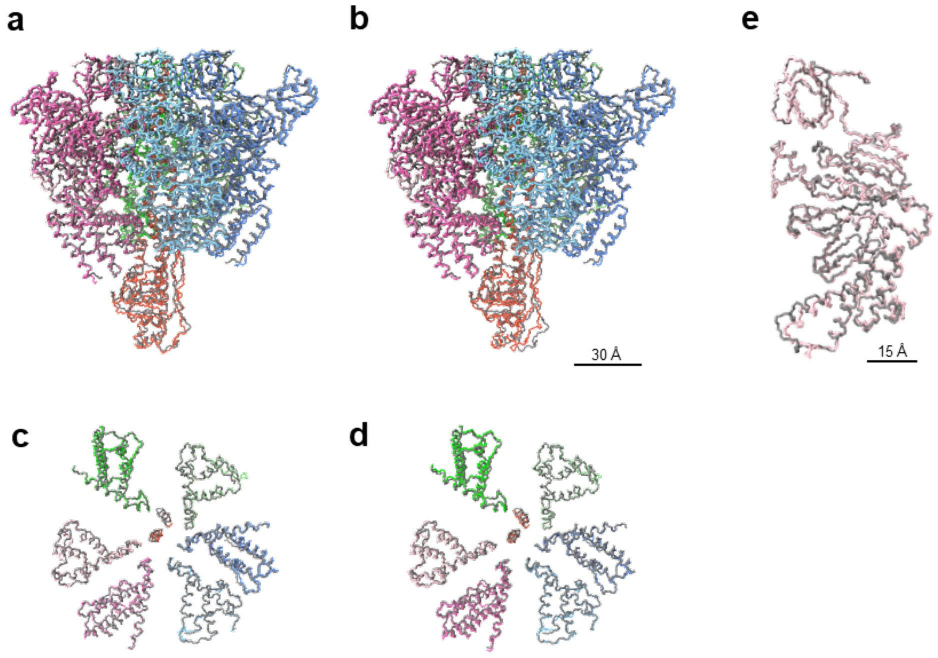

Supplementary Figure 5. Structural comparison of the $V_{nucfree}$, V_{3nuc}, and the ADP inhibited state of V/A-ATPase. **(a-d)** Comparison of overall and CHB structures of the $V_{nucfree}$ and the inhibited state (PDBID: 6QUM) **(a, c)**, and the V_{3nuc} state1-1 and the inhibited state **(b, d)**. **e,** Comparison of the B_{semi} subunits of $V_{nucfree}$ and V_{3nuc}. The structures are superimposed on the β-barrel domain (A subunit 1-70 a.a).

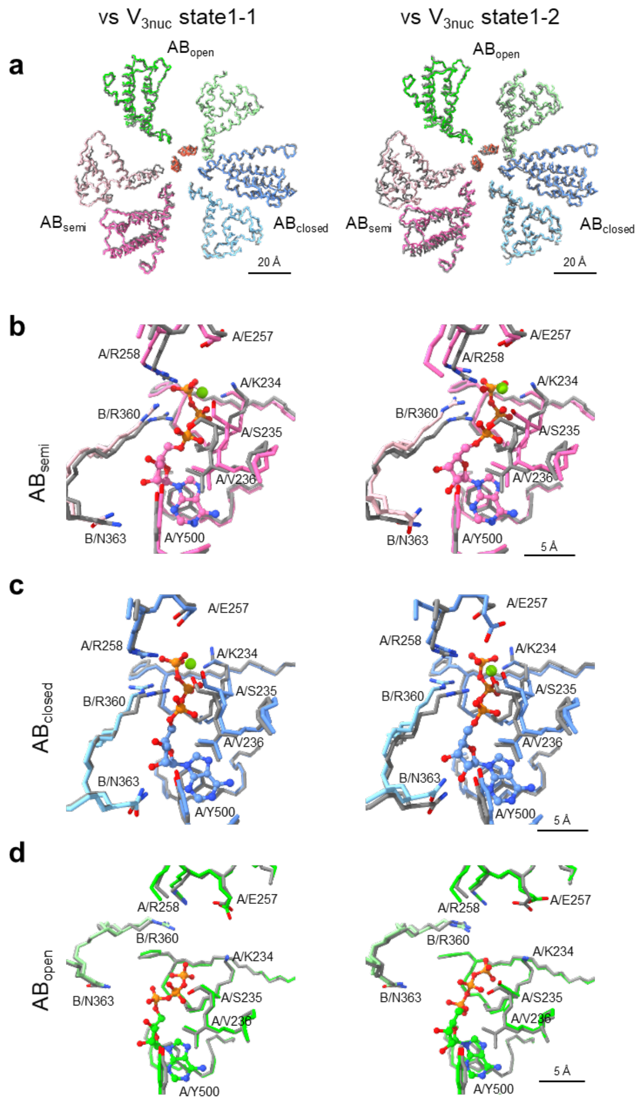

Supplementary Figure 6. Structural Comparison of $V_{nucfree}$ and V_{3nuc}. Comparison of the three catalytic sites of V_{3nuc} and $V_{nucfree}$. The structures are superimposed on the β barrel domain of the three A subunits (A:1-70 a.a.). **(a)** Comparison of the CHB. **(b-d)** Comparison of the catalytic site of AB_{semi} **(b)**, AB_{closed} **(c)**, and AB_{open} **(d)**. *Left panels;* $V_{nucfree}$ state1 (gray) vs V_{3nuc} state1-1 (colored). *Right panels;* $V_{nucfree}$ state1 (gray) *vs* V_{3nuc} state1-2 (colored).

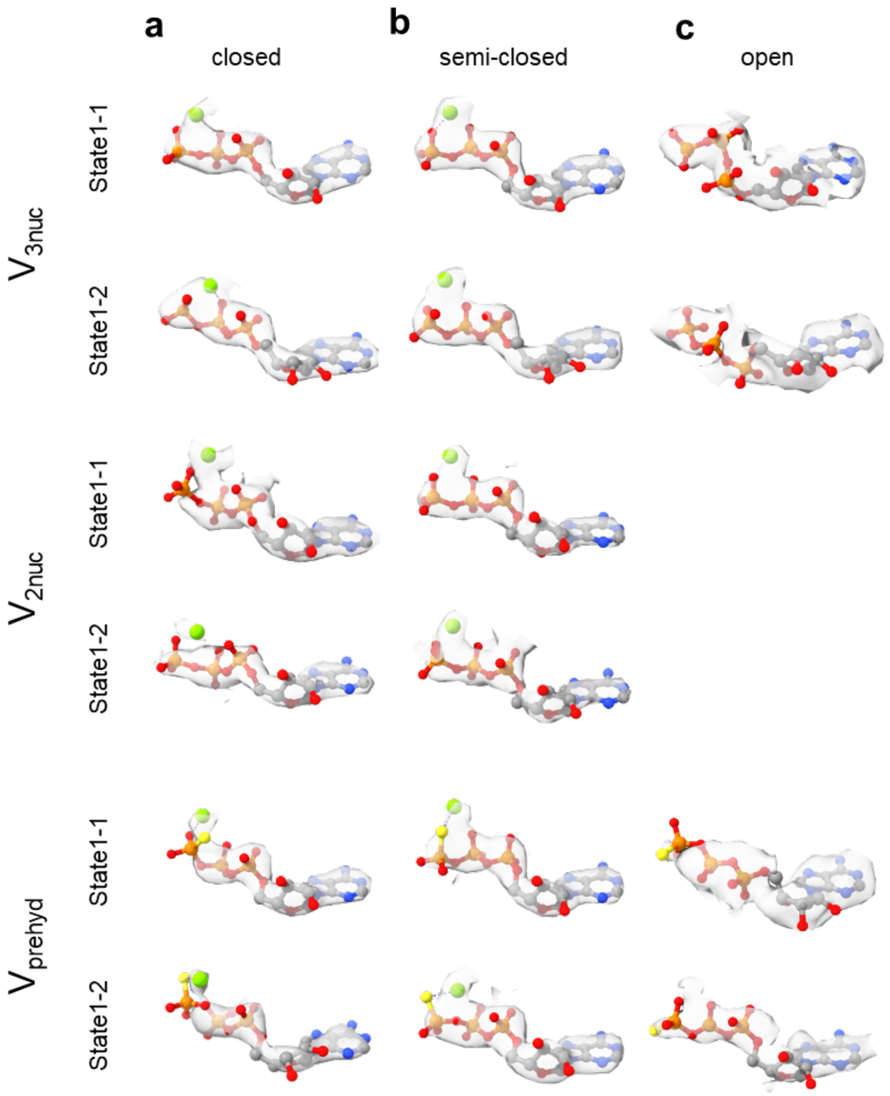

Supplementary Figure 7. EM density of bound nucleotides at each catalytic site. The densities corresponding to nucleotides and Mg ions are shown as semi-transparent surfaces. ATP and Mg ions are represented as ball-and-stick and spheres respectively. The catalytic sites of AB_{closed} **(a)**, AB_{semi} **(b)**, and AB_{open} **(c)**.

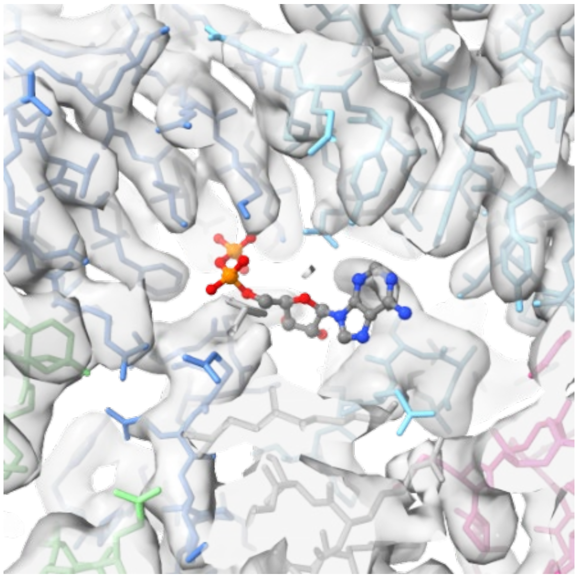

Supplementary Figure 8. No obvious density for an inhibitory nucleotide. The previously described structure containing ADP at the tip of the D subunit (PDBID: 6QUM) is fitted into the density map of V_{3nuc} state1-1 from this study.   There is no density corresponding to the inhibitory ADP.

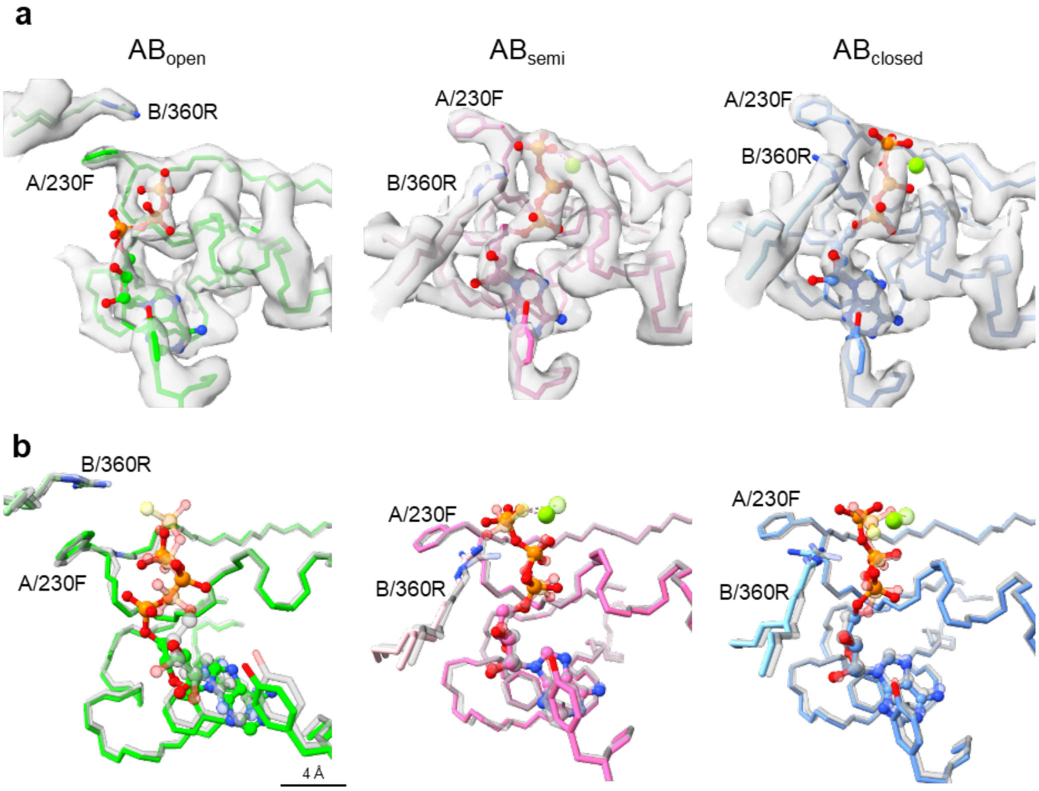

Supplementary Figure 9. Structural change of A/F230 in V_{prehyd} and V_{3ATP}. a, Magnified views of the three catalytic sites, AB_{open} (*left*), AB_{semi} (*middle*), and AB_{closed} (*right*) of V_{3nuc} state1-1. The chains are shown as sticks. ATP and Mg ions are shown in ball-and-stick and sphere representation, respectively. The density map is represented as a semi-transparent surface. **b**, Comparison of the catalytic sites between V_{3nuc} state1-1 (color) and V_{prehyd} state1-1 (grey).

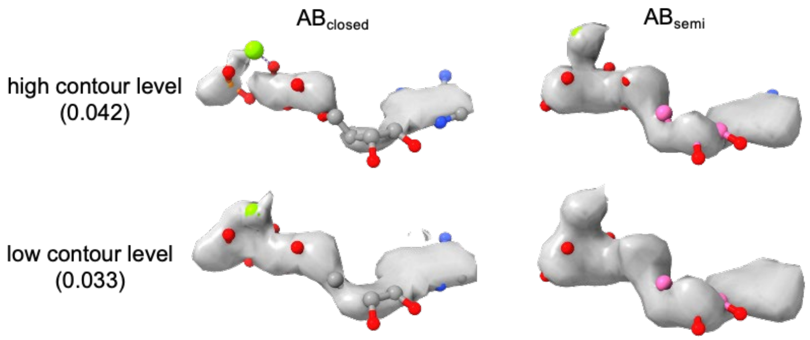

Supplementary Figure 10. EM density of ATP or ADP and P*i* in AB$_{closed}$ and AB$_{semi}$ of V$_{3nuc}$. The density corresponding to the bound nucleotides is represented as solid surfaces; AB$_{closed}$ (*left*) and AB$_{semi}$ (*right*). The contour levels are set to relatively high (*upper*) and low (*lower*). The nucleotides and magnesium ions are shown as balls and sticks.

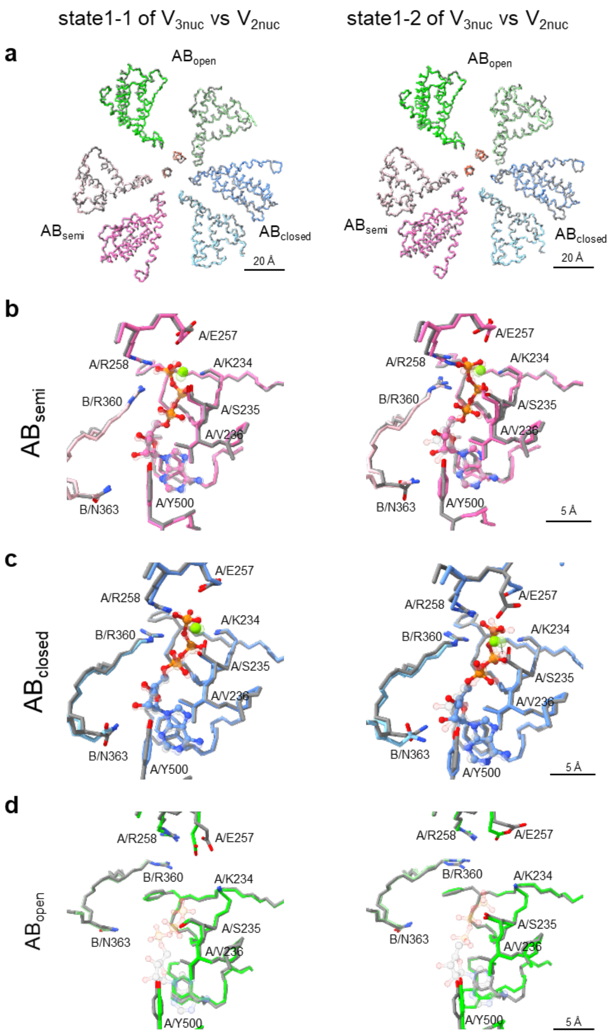

Supplementary Figure 11. Structural Comparison of V_{3nuc} and V_{2nuc}. Comparison of three catalytic sites between V_{3nuc} and V_{2nuc}. The structures are superimposed on the β barrel domain of the three A subunits (A:1-70 a.a.). **(a)** Comparison of the CHB. **(b-d)** Comparison of the catalytic site of AB_{semi} **(b)**, AB_{closed} **(c)**, and AB_{open} **(d)**. *Left panels;* V_{3nuc} state1-1 (gray) vs V_{2nuc} state1-1 (colored). *Right panels;* V_{3nuc} state1-2 (gray) vs V_{2nuc} state1-2 (colored).

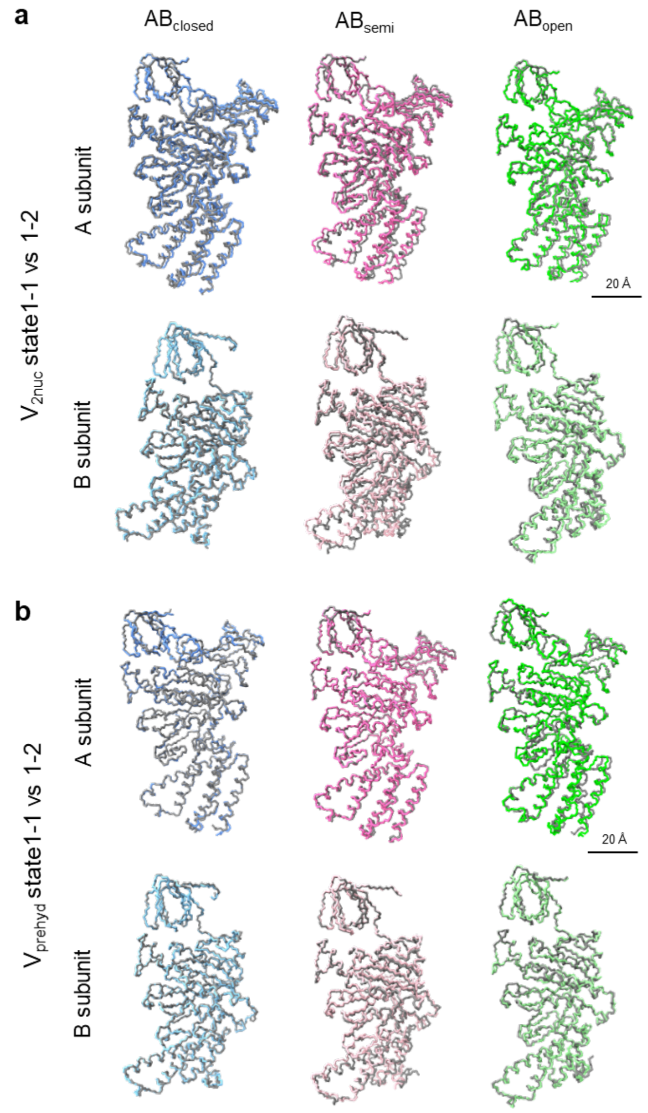

Supplementary Figure 12. Structural comparisons of each A and B subunit of state1-1 and state 1-2 of V_{2nuc} and V_{prehyd}. Each subunit was superimposed on the β-barrel domain (A subunit 1-70 a.a., B subunit 1-70 a.a.). **a**, Comparisons between state1-1 (gray) and state1-2 (colored) of V_{2nuc}. **b**, Comparisons between state1-1 (gray) and state1-2 (colored) of V_{prehyd}. The structures are shown as wire models.

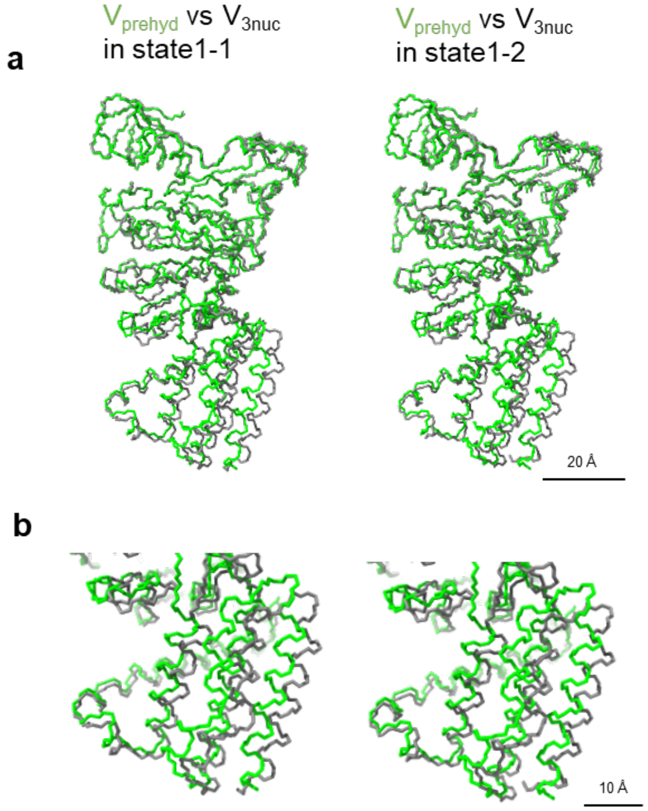

Supplementary Figure 13. Structural Comparison of A_{open} of V_{3nuc} and V_{prehyd}. The A_{open} subunit in V_{prehyd} state1-2 was compared with that of V_{3nuc} state1-1 and 1-2. The structures are represented as wire models, and are superimposed on the β barrel domain (A:1-70 a.a.). The subunits from V_{3nuc} and V_{prehyd} are colored in gray and green, respectively. **a**, Overall comparison with V_{3nuc} state1-1 (*left*) and state1-2 (*right*). **b**, Enlarged images of CHB shown in (A). RMSD values for the superpositions are summarized in Table S2.

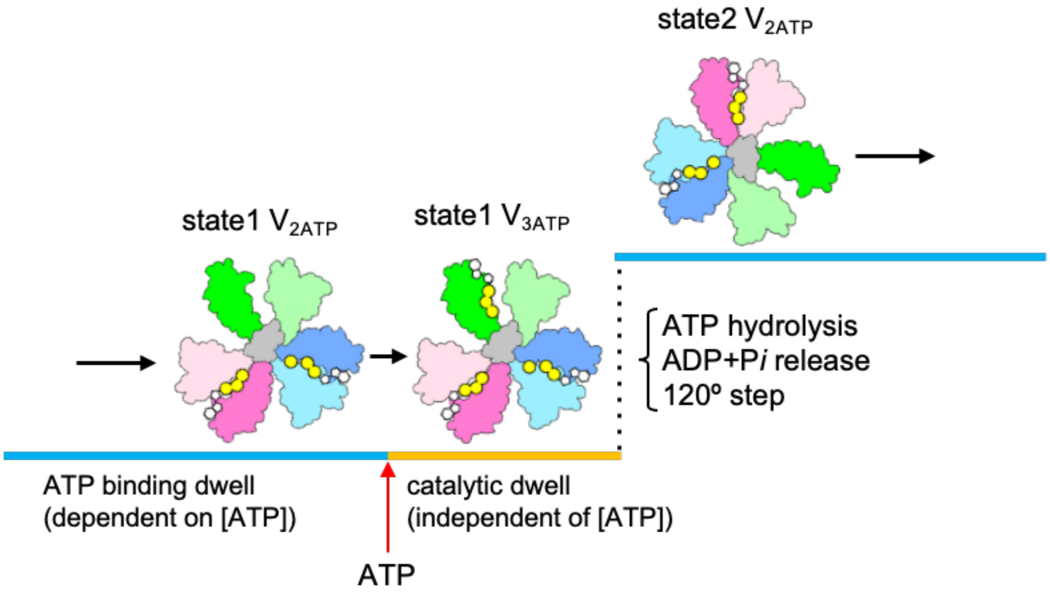

Supplementary Figure 14. An apparent ATP binding dwell due to awaiting both ATP binding and catalytic events. Schematic representation of the catalytic cycle of V/A-ATPase during the structural transition from state1 to state2. Following ATP binding, three catalytic processes, ATP hydrolysis in AB_{semi}, zippering in AB_{open} and product release in AB_{closed}, occur with the 120° step of the DF shaft.

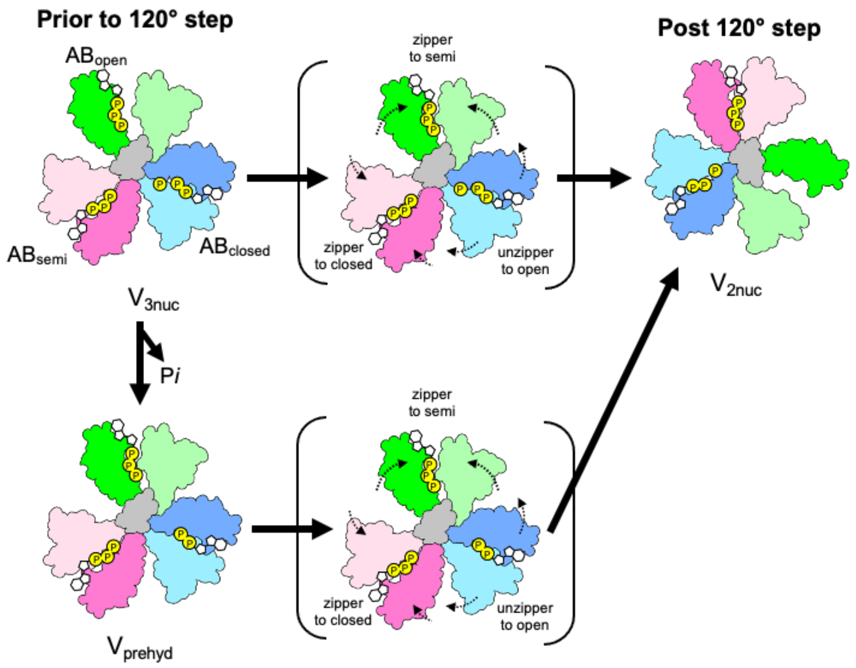

Supplementary Figure 15. Alternative pathway of V/A-ATPase rotation. Schematic models of AB_{open}, AB_{semi}, and AB_{closed} are shown in green, pink, and blue, respectively. The coiled coil region of the D subunit in contact with A_3B_3 is shown in grey. The V_{prehyd} corresponds to the structure in which phosphate is released from AB_{closed}. The catalytic events of the three AB dimers of V_{prehyd} occur simultaneously as in V_{3nuc}, resulting in structural transition of V_{prehyd} to V_{2nuc}.

Supplementary Table 1. Number of bound nucleotides of V/A-ATPase with or without nucleotide removal treatment.

	WT V/A-ATPase		TSSA V/A-ATPase	
	non-treated	treated	non-treated	Treated
ATP[1]	1.9	1.4	0.27	<0.01
ADP[1]	0.73	0.3	<0.01	<0.01
ATPase activity (s^{-1})	<1	5	45	51

[1]Number of binding nucleotides per enzyme.

Supplementary Table 2. RMSD values for A subunits of each state. The subunits were superimposed on the β-barrel domain (1-70 a.a), then the values for the back bone (444-573 a.a) were calculated using UCSF Chimera software. The lowest value in each row is indicated in bold.

(Å)

	444-573 (bb only)	V_{3nuc} state1-1	V_{3nuc} state1-2	V_{2nuc} state1-1	V_{2nuc} state1-2	V_{prehyd} state1-1	V_{prehyd} state1-2	$V_{nucfree}$ State1-1	$V_{nucfree}$ State1-2
A_{open}	V_{3nuc} state1-1		1.603	**0.534**	1.721	1.633	4.094	0.575	1.579
	V_{3nuc} state1-2	1.603		1.283	**0.485**	1.550	3.962	1.440	0.544
	V_{2nuc} state1-1	**0.534**	1.283		1.329	1.455	4.042	0.423	1.253
	V_{2nuc} state1-2	1.721	**0.485**	1.329		1.549	4.104	1.485	0.389
	V_{prehyd} state1-1	1.633	1.550	**1.455**	1.549		3.480	1.614	1.522
	V_{prehyd} state1-2	4.094	3.962	4.042	4.104	**3.48**		4.305	4.138
	$V_{nucfree}$ state1-1	0.575	1.440	**0.423**	1.485	1.614	4.305		1.278
	$V_{nucfree}$ state1-2	1.579	0.544	1.253	**0.389**	1.522	4.138	1.278	
A_{semi}	V_{3nuc} state1-1		2.306	**0.369**	2.603	1.576	1.990	0.602	2.041
	V_{3nuc} state1-2	2.306		2.081	**0.383**	1.855	1.365	1.936	0.548
	V_{2nuc} state1-1	**0.369**	2.081		2.390	1.413	1.783	0.464	1.789
	V_{2nuc} state1-2	2.603	**0.383**	2.39		2.092	1.524	2.206	0.720
	V_{prehyd} state1-1	1.576	1.855	1.413	2.092		**0.844**	1.530	1.759
	V_{prehyd} state1-2	1.99	1.365	1.783	1.524	**0.844**		1.733	1.363
	$V_{nucfree}$ state1-1	0.602	1.936	**0.464**	2.206	1.503	1.733		1.664
	$V_{nucfree}$ state1-2	2.041	**0.548**	1.789	0.720	1.759	1.363	1.664	
A_{closed}	V_{3nuc} state1-1		1.714	**0.278**	1.691	1.339	1.403	0.578	1.685
	V_{3nuc} state1-2	1.714		1.642	**0.292**	2.262	2.095	1.496	0.457
	V_{2nuc} state1-1	**0.278**	1.642		1.632	1.307	1.359	0.487	1.594
	V_{2nuc} state1-2	1.691	**0.292**	1.632		2.289	2.119	1.472	0.382
	V_{prehyd} state1-1	1.339	2.262	1.307	2.289		**0.381**	1.428	2.325
	V_{prehyd} state1-2	1.403	2.095	1.359	2.119	**0.381**		1.395	2.155
	$V_{nucfree}$ state1-1	0.578	1.496	**0.487**	1.472	1.428	1.395		1.413
	$V_{nucfree}$ state1-2	1.685	0.457	1.594	**0.382**	2.325	2.155	1.413	

Supplementary Table 3. RMSD values for B subunits of each state. The subunits were superimposed on the β-barrel domain (1-70 a.a), then the values for the back bone (374-446 a.a) were calculated using UCSF Chimera software. The lowest value in each row is indicated in bold.

(Å)

	374-446 (bb only)	V_{3nuc} state1-1	V_{3nuc} state1-2	V_{2nuc} state1-1	V_{2nuc} state1-2	V_{prehyd} state1-1	V_{prehyd} state1-2	$V_{nucfree}$ state1-1	$V_{nucfree}$ state1-2
B_{open}	V_{3nuc} state 1-1		1.531	**0.314**	1.310	1.168	1.200	0.346	1.309
	V_{3nuc} state 1-2	1.531		1.607	**0.477**	2.118	1.786	1.714	0.439
	V_{2nuc} state1-1	**0.314**	1.607		1.356	1.197	1.280	0.333	1.337
	V_{2nuc} state1-2	1.31	**0.477**	1.356		1.967	1.682	1.487	0.325
	V_{prehyd} state1-1	1.168	2.118	1.197	1.967		**0.535**	1.257	2.037
	V_{prehyd} state1-2	1.2	1.786	1.28	1.682	**0.535**		1.357	1.770
	$V_{nucfree}$ state1-1	0.346	1.714	**0.333**	1.487	1.257	1.357		1.488
	$V_{nucfree}$ state1-2	1.309	0.439	1.337	**0.325**	2.037	1.770	1.488	
B_{semi}	V_{3nuc} state1-1		3.647	**0.517**	3.594	1.992	3.268	0.803	3.240
	V_{3nuc} state1-2	3.647		3.561	**0.526**	2.755	2.161	3.906	1.014
	V_{2nuc} state1-1	**0.517**	3.561		3.512	1.885	3.142	0.606	3.109
	V_{2nuc} state1-2	3.594	**0.526**	3.512		2.732	2.295	3.840	0.757
	V_{prehyd} state1-1	1.992	2.755	1.885	2.732		2.096	2.240	2.465
	V_{prehyd} state1-2	3.268	2.161	3.142	2.295	**2.096**		3.464	2.206
	$V_{nucfree}$ state1-1	0.803	3.906	**0.606**	3.840	2.240	3.464		3.405
	$V_{nucfree}$ state1-2	3.240	1.014	3.109	**0.757**	2.465	2.206	3.405	
B_{closed}	V_{3nuc} state1-1		1.032	**0.352**	1.152	1.144	1.345	0.468	1.045
	V_{3nuc} state1-2	1.032		1.155	**0.369**	1.364	1.240	1.172	0.419
	V_{2nuc} state1-1	**0.352**	1.155		1.219	1.228	1.427	0.419	1.073
	V_{2nuc} state1-2	1.152	**0.369**	1.219		1.451	1.266	1.244	0.422
	V_{prehyd} state1-1	1.144	1.364	1.228	1.451		**0.542**	1.342	1.546
	V_{prehyd} state1-2	1.345	1.24	1.427	1.266	**0.542**		1.495	1.409
	$V_{nucfree}$ state1-1	0.468	1.172	**0.419**	1.244	1.342	1.495		1.096
	$V_{nucfree}$ state1-2	1.045	**0.419**	1.073	0.422	1.546	1.409	1.096	

Supplementary Table 4. Cryo-EM data collection, refinement and validation statistics for nucleotide-free V/A-ATPase.

	State1			State2		State3
	V_oV_1	V_1EG state1-1	V_1EG state1-2	V_oV_1	V_1EG	V_oV_1
EMDB ID	31841	31842	31843	31844	31845	31846
PDB ID		7VAI	7VAJ		7VAK	
**Data collection and processing**						
Magnification	81,000x					
EM & Voltage (kV)	Titan Krios, 300					
Total dose (e-/Å^2)	50					
Pixel size (Å/pix)	0.88					
Defocus range (µm)	-0.8 to -2.0					
Symmetry imposed	C1					
Initial particle #	4,354,341					
Final Particle #	68,290	39,902	24,103	12,770	12,770	5,188
Map resolution (Å)	3.1	3.1	3.1	4.7	4.1	6.3
FSC threshold	0.143					
**Refinement**						
Initial model used (PDBID)	-	6QUM	This study	-	This study	-
Model resolution (Å)	-	3.1 (masked)	3.1 (masked)	-	4.3 (masked)	-
FSC threshold	-	0.5	0.5	-	0.5	-
Model composition						
Nonhydrogen atoms	-	29,462	29,462	-	29,462	-
Protein residues	-	3,788	3,788		3,788	-
Ligands	-	0	0		0	-
R.m.s deviations						
Bond length (Å)	-	0.011	0.10		0.003	-
Bond Angles (°)	-	0.814	0.779		0.549	-
**Validation**						
MolProbity score	-	1.71	1.74		1.64	-
EMRinger score		3.41	2.98		1.59	-
Clashscore	-	6.94	7.27		8.89	-
Rotamer outlier (%)	-	0.03	0.05		0.00	-
CaBALM outlierl (%)		2.41	2.41		2.01	-
Ramachandran plot						
Favored (%)	-	95.32	95.14		97.05	-
Allowed (%)	-	4.65	4.81		2.95	-
Disallowed (%)	-	0.03	0.05		0.00	-

Supplementary Table 5. Cryo-EM data collection, refinement and validation statistics for V/A-ATPase obtained under 6 mM ATP condition.

	State1-1		State1-2	
	V_oV_1	V_1EG	V_oV_1	V_1EG
EMDB ID	31847	31849	31848	31850
PDB ID		7VAL		7VAM
**Data collection and processing**				
Magnification	81,000x			
EM & Voltage (kV)	Titan Krios, 300			
Total dose (e-/Å^2)	50			
Pixel size (Å/pix)	0.88			
Defocus range (μm)	-0.8 to -2.0			
Symmetry imposed	C1			
Initial particle #	2,300,834			
Final Particle #	40,831		28,801	
Map resolution (Å)	3.5	3.1	3.6	3.2
FSC threshold	0.143			
**Refinement**				
Initial model used (PDBID)	-	This study	-	This study
Model resolution (Å)	-	3.0 (masked)	-	3.2 (masked)
FSC threshold	-	0.5	-	0.5
Model composition				
Nonhydrogen atoms	-	29,557	-	29,557
Protein residues	-	3,788	-	3,788
Ligands	-	2 ATP, ADP, Pi, 2 MG	-	2 ATP, ADP, Pi, 2 MG
Bond length (Å)	-	0.003	-	0.004
Bond Angles (°)	-	0.547	-	0.630
**Validation**				
MolProbity score	-	1.12	-	1.17
EMRinger score	-	3.92	-	3.36
Clashscore	-	3.32	-	3.82
Rotamer outlier (%)	-	0.00	-	0.00
CaBALM outlierl (%)	-	1.36	-	1.52
Ramachandran plot				
Favored (%)	-	98.51	-	98.59
Allowed (%)	-	1.49	-	1.41
Disallowed (%)	-	0.00	-	0.00

Supplementary Table 6. Cryo-EM data collection, refinement and validation statistics for V/A-ATPase obtained under 6 mM ATP condition.

	State2-1		State2-2	
	V_oV_1	V_1EG	V_oV_1	V_1EG
EMDB ID	31851	31853	31852	31854
PDB ID		7VAN		7VAO
**Data collection and processing**				
Magnification	81,000x			
EM & Voltage (kV)	Titan Krios, 300			
Total dose (e-/Å^2)	50			
Pixel size (Å/pix)	0.88			
Defocus range (μm)	-0.8 to -2.0			
Symmetry imposed	C1			
Initial particle #	2,300,834			
Final Particle #	36,693		12,087	
Map resolution (Å)	3.3	3.0	4.0	3.4
FSC threshold	0.143			
**Refinement**				
Initial model used (PDBID)	-	This study	-	This study
Model resolution (Å)	-	3.0 (masked)	-	3.4 (masked)
FSC threshold	-	0.5	-	0.5
Model composition				
Nonhydrogen atoms	-	29,557	-	29,557
Protein residues	-	3,788	-	3,788
Ligands	-	2 ATP, ADP, Pi, 2 MG	-	2 ATP, ADP, Pi, 2 MG
Bond length (Å)	-	0.005	-	0.004
Bond Angles (°)	-	0.571	-	0.552
**Validation**				
MolProbity score	-	1.25	-	1.30
EMRinger score	-	3.81	-	3.03
Clashscore	-	3.69	-	4.48
Rotamer outlier (%)	-	0.00	-	0.00
CaBALM outlierl (%)		1.44	-	1.47
Ramachandran plot				
Favored (%)	-	97.53	-	97.66
Allowed (%)	-	2.47	-	2.34
Disallowed (%)	-	0.00	-	0.00

Supplementary Table 7. Cryo-EM data collection, refinement and validation statistics for V/A-ATPase obtained under 6 mM ATP condition.

	State3-1		State3-2	
	V_oV_1	V_1EG	V_oV_1	V_1EG
EMDB ID	31855	31857	31856	31858
PDB ID		7VAP		7VAQ
**Data collection and processing**				
Magnification	81,000x			
EM & Voltage (kV)	Titan Krios, 300			
Total dose (e-/Å^2)	50			
Pixel size (Å/pix)	0.88			
Defocus range (μm)	-0.8 to -2.0			
Symmetry imposed	C1			
Initial particle #	2,300,834			
Final Particle #	31,631		5,300	
Map resolution (Å)	3.3	3.0	4.5	3.6
FSC threshold	0.143			
**Refinement**				
Initial model used (PDBID)	-	This study	-	This study
Model resolution (Å)	-	3.1 (masked)	-	3.6 (masked)
FSC threshold	-	0.5	-	0.5
Model composition				
Nonhydrogen atoms	-	29,557	-	29,557
Protein residues	-	3,788	-	3,788
Ligands	-	2 ATP, ADP, Pi, 2 MG	-	2 ATP, ADP, Pi, 2 MG
Bond length (Å)	-	0.004	-	0.003
Bond Angles (°)	-	0.526	-	0.0524
**Validation**				
MolProbity score	-	1.18	-	1.29
EMRinger score	-	3.49	-	2.51
Clashscore	-	3.86	-	5.44
Rotamer outlier (%)	-	0.00	-	0.00
CaBALM outlierl (%)	-	1.66	-	1.71
Ramachandran plot				
Favored (%)	-	97.95	-	98.19
Allowed (%)	-	2.05	-	1.81
Disallowed (%)	-	0.00	-	0.00

Supplementary Table 8. Cryo-EM data collection, refinement and validation statistics for V/A-ATPase obtained under 50 µM ATP condition.

	State1		
	V_oV_1	V_1EG state1-1	V_1EG state1-2
EMDB ID	31859	31860	31861
PDB ID		7VAR	7VAS
**Data collection and processing**			
Magnification	81,000x		
EM & Voltage (kV)	Titan Krios, 300		
Total dose (e-/Å^2)	50		
Pixel size (Å/pix)	0.88		
Defocus range (μm)	-0.8 to -2.0		
Symmetry imposed	C1		
Initial particle #	1,671,397		
Final Particle #	84,150	42,167	35,872
Map resolution (Å)	2.7	2.9	3.0
FSC threshold	0.143		
**Refinement**			
Initial model used (PDBID)	-	This study	This study
Model resolution (Å)	-	3.0 (masked)	3.1 (masked)
FSC threshold	-	0.5	0.5
Model composition			
Nonhydrogen atoms	-	29,529	29,517
Protein residues	-	3,787	3,787
Ligands	-	ATP, ADP 2 MG	ATP, ADP, 2 MG
Bond length (Å)	-	0.003	0.003
Bond Angles (°)	-	0.536	0.502
**Validation**			
MolProbity score	-	1.08	1.19
EMRinger score	-	3.92	3.52
Clashscore	-	2.93	4.10
Rotamer outlier (%)	-	0.06	0.13
CaBALM outlierl (%)	-	1.45	1.74
Ramachandran plot			
Favored (%)	-	98.83	98.14
Allowed (%)	-	1.17	1.86
Disallowed (%)	-	0.00	0.00

Supplementary Table 9. Cryo-EM data collection, refinement and validation statistics for V/A-ATPase obtained under 50 µM ATP condition.

	State2			State3	
	V_oV_1	V_1EG state2-1	V_1EG state2-2	V_oV_1	V_1EG
EMDB ID	31862	31863	31864	31865	31866
PDB ID		7VAT	7VAU		7VAV
**Data collection and processing**					
Magnification	81,000x				
EM & Voltage (kV)	Titan Krios, 300				
Total dose (e-/Å^2)	50				
Pixel size (Å/pix)	0.88				
Defocus range (μm)	-0.8 to -2.0				
Symmetry imposed	C1				
Initial particle #	1,671,397				
Final Particle #	48,316	28,883	11,908	26,525	
Map resolution (Å)	3.3	3.2	3.3	3.6	2.8
FSC threshold	0.143				
**Refinement**					
Initial model used (PDBID)	-	This study	This Study	-	This study
Model resolution (Å)	-	3.1 (masked)	3.5 (masked)	-	3.4 (masked)
FSC threshold	-	0.5	0.5	-	0.5
Model composition					
Nonhydrogen atoms	-	29,529	29,517	-	29,529
Protein residues	-	3,787	3,787	-	3,787
Ligands	-	ATP, ADP, 2 MG	ATP, ADP, 2 MG	-	ATP, ADP, 2 MG
Bond length (Å)	-	0.003	0.007	-	0.002
Bond Angles (°)	-	0.507	0.670	-	0.470
**Validation**					
MolProbity score	-	1.16	1.39	-	1.18
EMRinger score	-	3.36	3.02	-	3.20
Clashscore	-	3.69	5.14	-	3.96
Rotamer outlier (%)	-	0.00	0.00	-	0.00
CaBALM outlierl (%)	-	1.69	1.79	-	1.47
Ramachandran plot					
Favored (%)	-	98.19	97.39	-	98.54
Allowed (%)	-	1.81	2.61	-	1.46
Disallowed (%)	-	0.00	0.00	-	0.00

Supplementary Table 10. Cryo-EM data collection, refinement and validation statistics for V/A-ATPase obtained under 4 mM ATPγS condition.

	State1		
	V_oV_1	State1-1 V_1EG	State1-2 V_1EG
EMDB ID	31867	31868	31869
PDB ID		7VAW	7VAX
Data collection and processing			
Magnification	60,000x		
EM & Voltage (kV)	CRYOARM300, 300		
Total dose (e-/Å^2)	50		
Pixel size (Å/pix)	0.81		
Defocus range (μm)	-0.8 to -2.0		
Symmetry imposed	C1		
Initial particle #	4,677,284		
Final Particle #	140,014	45,066	49,606
Map resolution (Å)	2.7	2.7	2.9
FSC threshold	0.143		
Refinement			
Initial model used (PDBID)	-	This study	This study
Model resolution (Å)	-	2.9 (masked)	3.0 (masked)
FSC threshold	-	0.5	0.5
Model composition			
Nonhydrogen atoms	-	29,557	29,557
Protein residues	-	3,788	3,788
Ligands	-	2 ATPγS, ADP, 2 MG	2 ATPγS, ADP, 2 MG
Bond length (Å)	-	0.004	0.002
Bond Angles (°)	-	0.566	0.492
Validation			
MolProbity score	-	1.23	1.27
EMRinger score	-	3.97	3.26
Clashscore	-	2.93	5.00
Rotamer outlier (%)	-	0.03	0.03
CaBALM outlierl (%)	-	1.74	1.55
Ramachandran plot			
Favored (%)	-	98.57	97.98
Allowed (%)	-	1.43	2.02
Disallowed (%)	-	0.00	0.00

Supplementary Table 11. Cryo-EM data collection, refinement and validation statistics for V/A-ATPase obtained under 4 mM ATPγS condition.

	State2		State3	
	V_oV_1	V_1EG	V_oV_1	V_1EG
EMDB ID	31870	31871	31872	31873
PDB ID		7VAY		7VB0
**Data collection and processing**				
Magnification	60,000x			
EM & Voltage (kV)	CRYOARM300, 300			
Total dose (e-/Å^2)	50			
Pixel size (Å/pix)	0.81			
Defocus range (μm)	-0.8 to -2.0			
Symmetry imposed	C1			
Initial particle #	4,677,284			
Final Particle #	35,300		22,646	
Map resolution (Å)	3.3	3.3	3.6	3.6
FSC threshold	0.143			
**Refinement**				
Initial model used (PDBID)	-	This study	-	This Study
Model resolution (Å)	-	3.3 (masked)	-	3.5 (masked)
FSC threshold	-	0.5	-	0.5
Model composition				
Nonhydrogen atoms	-	29,557	-	29,557
Protein residues	-	3,788	-	3,788
Ligands	-	2 ATPγS, ADP, 2 MG	-	2 ATPγS, ADP, 2 MG
Bond length (Å)	-	0.004	-	0.003
Bond Angles (°)	-	0.572	-	0.534
**Validation**				
MolProbity score	-	1.45	-	1.34
EMRinger score	-	2.87	-	2.86
Clashscore	-	6.16	-	6.11
Rotamer outlier (%)	-	0.00	-	0.00
CaBALM outlierl (%)	-	1.82	-	1.76
Ramachandran plot				
Favored (%)	-	97.42	-	98.09
Allowed (%)	-	2.58	-	1.91
Disallowed (%)	-	0.00	-	0.00
