# Supplementary material for: Structural snapshots of V/A-ATPase reveal the rotary catalytic mechanism of rotary ATPases
Source: Nat Commun. 2022 Mar 8;13:1213. doi: 10.1038/s41467-022-28832-5 (PMC8904598; doi:10.1038/s41467-022-28832-5)
Supplement: Supplementary file 3 — Description of Additional Supplementary Files [file 41467_2022_28832_MOESM3_ESM.pdf]

Description of Additional Supplementary Files

File name: Supplementary Movie 1

Description: **Structural transition from state1 of $V_{nucfree}$ to state2 of V_{2nuc}.** The state1 of $V_{nucfree}$ is thermally fluctuating between state1-1 and state1-2. This thermal fluctuation also occurs in V_{3nuc} and V_{2nuc}. The $V_{nucfree}$ in ground state is activated by the binding of ATP to the catalytic sites. In V_{2nuc} awaiting ATP binding, binding of ATP onto AB_{open} produces state1 of V_{3nuc}. In V_{3nuc}, the catalytic events in three AB dimers occurs simultaneously with 120˚ step of DF shaft, resulting in structural transition of state1of V_{3nuc} to state2 of V_{2nuc}.

File name: Supplementary Movie 2

Description: **Rotation of V_1 moiety driven by ATP hydrolysis.** The state1 of V_{2nuc} is thermally fluctuating between state1-1 and state1-2. The binding of ATP onto AB_{open} produces state1 of V_{3nuc}.The catalytic events in three AB dimers in V_{3nuc} occurs simultaneously with 120˚ step of DF shaft.
